# ZIKA Virus, an Emerging Arbovirus in India: A Glimpse of Global Genetic Lineages

**DOI:** 10.3390/microorganisms13030544

**Published:** 2025-02-27

**Authors:** Paramasivan Rajaiah, Bhavna Gupta, Muniyaraj Mayilsamy

**Affiliations:** ICMR-Vector Control Research Centre, 4, Sarojini Street, Chinna Chokkikulam, Madurai 625 002, India; bhavna.g@icmr.gov.in (B.G.); muniyaraj.m@icmr.gov.in (M.M.)

**Keywords:** ZIKA virus, ZIKA fever, microcephaly, Guillain–Barre syndrome, African lineage, Asian lineage

## Abstract

ZIKA fever (ZIKAF) is an emerging mosquito-borne flavivirus illness in humans. Regarding the etiological agent, ZIKA virus (ZIKAV), though it is known to be distributed in the tropics, causing sporadic cases, its rapid global expansion with pandemic potential has raised global concern. Due to its abrupt emergence in South American countries, the Caribbean, and the Americas, the WHO declared ZIKA a public health emergency of international concern in 2016. ZIKAV usually causes mild infections; however, its recent unusual presentations of Guillen–Barré syndrome in adults and microcephaly in newborn babies of ZIKAV-infected mothers in Brazil has caused concern among global public health authorities. Certain mutations on virus genomes have been found to be correlated with clinical severity, and its unusual transmission routes through sexual and blood transfusions emphasize the necessity for understanding its virological determinants and impact. Its abrupt re-emergence in India (2018–2019), particularly in Gujarat (2016), Tamil Nadu (2017), Uttar Pradesh (2021), Maharashtra, Kerala (2021), and Karnataka (2023), has indicated the need for urgent measures to strengthen surveillance systems and design effective prevention and control measures in this country. Given the global concern around ZIKAV, here, we reviewed current knowledge about global ZIKAV genetic lineages vis à vis the situation in India and discussed future priorities for ZIKAV research in India for effectively designing control strategies.

## 1. Introduction

Vector-borne diseases are considered an emerging public health concern globally. The term “ZIKA” has recently drawn widespread attention worldwide because of its abrupt global emergence and rapidly expanding potential in new areas. ZIKA virus (ZIKAV) is transmitted to humans by *Aedes aegypti* mosquitoes. In humans, the virus usually causes a subclinical or mild febrile illness called ZIKA fever (ZIKAF). ZIKAF clinically presents with a mild febrile undifferentiated illness and is often considered to be self-limiting. However, recent reports have revealed severe clinical manifestations, such as microcephaly in newborn babies delivered to ZIKAV-infected mothers and Guillen–Barré syndrome (GBS), a serious neurological disorder that warns of a looming global public health emergency. No effective commercial vaccines or treatment strategies are available to prevent and control ZIKAV. Controlling the vector is the only way to effectively control this disease.

ZIKA virus (ZIKAV) was first recovered from a sentinel rhesus macaque in ZIKA forest in Entebbe, Uganda, Africa, in 1947 [1]. In 1948, the virus was subsequently isolated from *Aedes africanus* mosquitoes in Uganda. The first case of human infection due to ZIKAV was detected in 1952 in Tanzania, Africa. Later, the virus was found to be involved in causing outbreaks of human infection on Yap Island, Micronesia, in 2007 [2], French Polynesia in 2013–2014 [3], and Brazil (2015) [4]. Besides Africa, ZIKAV is widely distributed in several Asian countries, including Vietnam, the Philippines, Thailand, Cambodia, Indonesia, Singapore, Japan, and the Americas [5].

**Figure 1:**
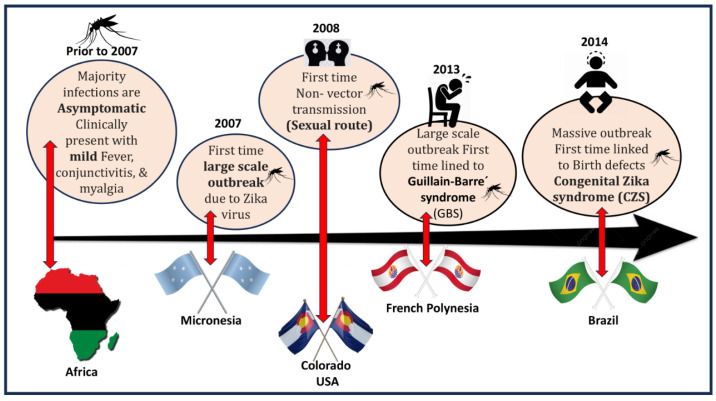
Illustration showing the time-scale events of ZIKAV clinical manifestations that have occurred in the affected areas.

Although the virus is transmitted to humans primarily through mosquito bites, recent reports of infection through sexual contact [6] indicate a potential non-vectorial, multimodal route of transmission, which indicates that suitable intervention strategies need to be developed. Moreover, transmission through contaminated blood and blood products [7] further complicates existing control measures. Acute ZIKAV infection occurs mainly through subclinical infection, and the CFR of acute cases is found to be very low [8]. However, the estimated risk of new people acquiring ZIKV infection is projected to exceed 1.3 billion by 2050 due to global warming [9]. An effective commercial vaccine or antiviral drug is presently lacking, and vector control methods are the only available option for prevention and control. Figure 1 shows the time-scale events of ZIKAV clinical manifestations that have occurred globally.

Identifying a ZIKAV infection is difficult due to its overlapping clinical presentations with other commonly occurring mosquito-transmitted infections, such as dengue and Chikungunya infections [10]. Additionally, serological cross-reactions among closely related pathogens further complicate diagnostic decisions in endemic settings. Highly specific and sensitive diagnostic assays are needed for the timely and accurate detection of ZIKAV infections in endemic areas to control them. Along with the accurate detection of viruses, the development of an effective vaccine is another important part of research on the prevention and control of this disease. Knowledge of the circulating serotypes/genotypes of ZIKAV in particular geographic areas is necessary, as this information can complement diagnostic and effective vaccine development efforts. Additionally, owing to ongoing evolutionary events, the acquisition of mutational substitutions in circulating genotypes may reflect the clinical outcomes of the disease, such as the efficient transmissibility of mutant viruses and an increase in neurovirulence. Also, understanding circulating genotypes is crucial for developing future control measures.

There are no approved vaccines available for the successful prevention and control of ZIKA virus infection. However, many vaccine development attempts are already underway, exploiting different platforms such as live attenuated viruses, inactivated viruses, virus-like particles (VLPs), protein- and peptide-based strategies, viral vectors, DNA- and mRNA-based platforms, etc. [11].

In India, although ZIKAV has been in circulation since 1947 [12], information on the circulating genotypes in this country needs to be obtained, and these genotypes need to be monitored in endemic areas and potential hotspots in the country.

## 2. Genomic Organization of ZIKAV

**Figure 2:**
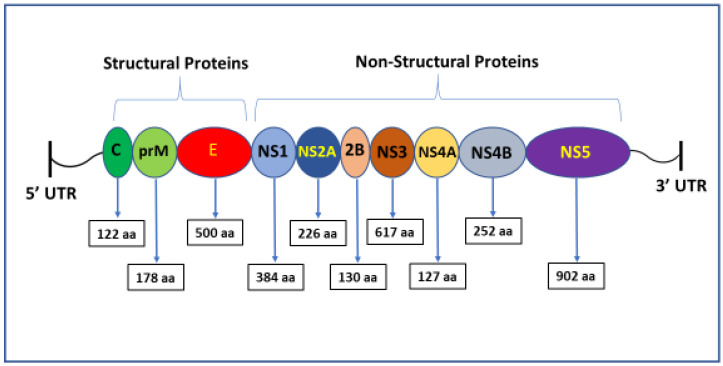
Genomic organization of the ZIKAV.

ZIKAV belongs to the Spondweni serogroup of arboviruses. As an enveloped RNA virus, its genome is about 11,000 bp in length and encodes three structural proteins (C, prM, and E) and seven non-structural proteins (NS1, NS2A, NS2B, NS3, NS4A, NS4B, and NS5) as a single polyprotein flanked by 5′ and 3′ untranslated regions (UTRs) (Figure 2). As a member of the genus flavivirus, the ZIKA virus genome also contains conserved dinucleotide complimentary terminal sequences (5′-AG … CU-3′) [13].

**Figure 3:**
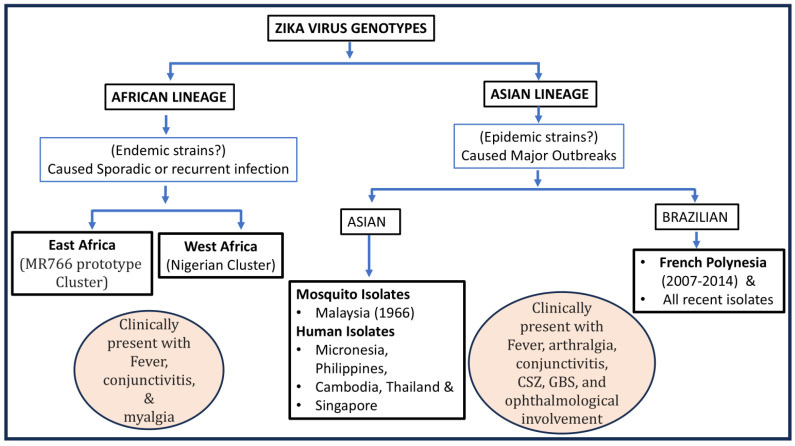
A schematic illustration of the circulating genotypes of ZIKAV around the world.

The virus genome has a methylated cap and a loop structure at its 5′ and 3′ termini, respectively [14]. As an RNA virus, the evolutionary rate of the Asian ZIKV lineage was estimated to be 7.26 × 10^−4^ substitutions per site per year (95% HPD: 6.28–8.19 × 10^−4^) [15]. Based on the analysis of the C and E gene sequences of the virus, ZIKAV is classified into two major genetic lineages: Asian and African. The strains recovered from different outbreaks were grouped among these two major lineages. The strains recovered from Southeast Asia, the Pacific, and the American Islands were grouped under Asian lineages. Strains circulating in East Africa and West Africa were grouped under the African lineage. Interestingly, lineages recovered from epidemics have been grouped under Asian lineages, which indicates the emergence and epidemic potential of these genotypes, whereas strains originating from the African lineage are usually found endemically, particularly in East and West Africa. These endemic lineages of African origin are usually not involved in outbreaks and cause mild infections. A global depiction of the distribution of genetic lineages of ZIKAV is shown in Figure 3.

ZIKA viruses were first detected in 1947 and are found circulating extensively in Africa and Asia, with substantial evolutionary changes. Various studies comparing the genomes of global ZIKAV lineages have classified them into African and Asian lineages, which are found to have very few amino acid changes in their genomes. However, in-depth analysis has revealed more genetic variants among Asian lineages than African lineage viruses [16,17]. Additionally, African and Asian lineages of the ZIKAV behave differently in terms of pathogenesis and laboratory characteristics [18].

## 3. A Global Picture of Genetic Lineages

The ZIKA virus causes geographically localized mild-to-moderate infections among humans. Various studies have documented the genetic dynamics of ZIKAV over time in different parts of the world.

Although ZIKAV has been present in Africa and Asia (Malaysia) since 1947 and 1966, respectively, the abrupt emergence of ZIKAV in Brazil in 2015 with serious microcephaly cases in newborns [19] attracted global attention for the development of urgent measures for its effective intervention. The massive outbreak of ZIKAV in Brazil (2015) was caused by Asian lineage viruses, which were probably introduced from the Pacific Islands, particularly from French Polynesia. Thus, ZIKAV spread to other parts of the globe, particularly to the Americas, from French Polynesia [20]. Studies suggest that after the detection of the Asian lineage of ZIKAV in Malaysia in 1966, it underwent continuous mutational changes throughout Southeast Asia and evolved into the most efficient type of ZIKAV with high outbreak potential, spread to FP, and caused massive outbreaks. From French Polynesia, the introduced ZIKAV, belonging to the Asian lineage, further spread to other parts of the world.

After the massive outbreak in Brazil (2015), a minor epidemic was recorded in Singapore in 2016 [21]. ZIKAV infection was subsequently detected in Malaysia, which has similar ecological conditions to Singapore and Brazil. Interestingly, despite the detection of ZIKAV among *A. aegypti* mosquitoes in Bentong Pahang, Malaysia, in 1966 [22], no outbreak of ZIKA has been recorded. The reason behind this mild form of the disease was found to be the existence of pre-immune antibodies in the local population and the circulation of local virus strains in the country [23]. This evidence indicates that compared to the Brazilian strains, the native strains of ZIKAV in Malaysia cause mild infections. Furthermore, understanding the virological dynamics of the circulating strains of ZIKAV and their interaction with local and exotic *Aedes* mosquitoes is important for developing future control efforts.

The ZIKA virus is an RNA virus similar to other mosquito-transmitted flaviviruses, such as the West Nile and JE viruses. It has undergone continuous evolutionary changes in endemic areas, and predicting its transmissibility and clinical dynamics based on these evolutionary events at the genetic level is difficult. The African lineages of ZIKAV are believed to cause mild infections; however, recent experimental evidence from appropriate cell lines, mosquitoes, and mouse models has shown that African lineage ZIKAV strains have greater transmissibility and pathogenicity than Asian lineage ZIKAV strains [24]. This new laboratory-based knowledge highlights that African ZIKAV lineages may give rise to potent outbreak strains. Adequate field-based data are needed in this area to confirm this trait to better understand its virological dynamics.

## 4. Mutation of ZIKAV and Its Impact on Phenotypes

Genomic analysis studies have revealed limited nucleotide divergence among the open reading frames (ORFs) of African (isolated from East Africa, Central Africa, and West Africa) and Asian (recovered in Southeast Asia, the Pacific Islands, and the Americas) ZIKAV strains [25].

Flaviviruses constantly undergo mutational substitutions, mainly due to their lack of proofreading activity, and exhibit alterations in virulence and tropism [26]. ZIKAV constantly undergoes mutational changes with a high rate of rapid mutation of about 10 mutations per year [27]; i.e., RNA mutates at a rate equivalent to about 0.01% each year. However, when the African and Asian lineages were compared, the African lineages were generally considered endemic, and the Asian lineages were found to have epidemic potential. Although ZIKAV was first detected in Africa (1947) and subsequently in Malaysia (1966), its geographical distribution is restricted. While comparing the original strain [MR766 (HQ234498)] and the subsequent variants (AY632535 and DQ859059) recovered from rhesus monkeys (Uganda, 1947), a low nucleotide variation of 0.4% of nucleotides encoding 0.6% of amino acid sequences was observed [28]. This is probably related to the low infection efficiency required to circulate among humans, which mostly causes subclinical infections. However, at a later stage, many studies documented the acquisition of mutations, which may have facilitated the rapid global spread of the virus, along with its altered pathology [29]. Moreover, experiments have shown the altered phenotypic expression of isolated or even minimal mutations in the viral genome [30].

Among ZIKA viruses, mutations vary between 12 and 25 substitutions per year [31]. As vector-borne infections, these mutational substitution events might have taken place in humans/vector mosquitoes and could have certain traits, such as increased transmissibility, increased virulence, and immune escape ability. Early isolates, i.e., the original isolates, were found to have between 9 and 30 mutations in a year. However, the recent outbreak isolates KU740184 (the GZ01/2016 strain isolated from a Chinese patient who returned from Venezuela in 2016) and KU 744693 (the VE-Ganxian isolate from China) presented 30 and 64 mutations per year, respectively [31]. This difference in mutational substitution between original and recent field-adapted outbreak isolates could be due to the availability of many human hosts and the opportunity to undergo rapid mutation; such mutations occurred mainly due to the continuous selective pressure exerted by hosts or vectors. Researchers have also reported differences in replication efficiency and the differential regulation of the host innate immune response between Asian and African lineages of ZIKAV [32]. Additionally, in the ZIKAV genome, mutational substitutions occur in almost all genes, including structural and non-structural genes, and their respective traits enhance phenotypic properties, with the emergence of fit genotypes capable of causing outbreaks [33]. This evidence indicates the possible emergence of more virulent strains of ZIKAV. A list of different substitutions that take place in the gene regions of ZIKAV and their effect on the phenotypic expression of the virus is provided in (Table 1).

During an in-depth analysis of the genetic details of various ZIKAV strains, ref. [34] found that 75 amino acids were different between pre-epidemic and epidemic strains of global ZIKAV isolates. Specifically, 15 substitutions have only been found in epidemic lineages of ZIKAV and not in pre-epidemic strains. Evidence has indicated the possible occurrence of recombination events between ZIKAV and the Spondweni virus, particularly in the NS2B coding region between the 4237 and 4528 nucleotide positions. The analysis also showed that nine nucleotide bases form a large bulge at SL1 in the epidemic ZIKAV strains, which resembled the SLII of the pre-epidemic strain. Further studies are needed to determine the clinical relevance of these changes in the epidemic strains of ZIKAV.

Reports on the genome analysis of ZIKAV have revealed successful nucleotide substitutions in structural and non-structural genes among the viruses analyzed. Among the structural coding proteins, amino acid substitutions in the C coding region (five amino acid substitutions: I113V, R101K, I110V, L27F, and N25S), PrM coding region (V262A, K246R, V1581, H157Y, A148P, K143E, S139N, 1125 V, and V153M), and domain III of the E region (V603I and D679E) have been detected in epidemic strains but not in pre-epidemic strains. Extensive analysis including more samples may elucidate the behavior of ZIKAV. The accumulated laboratory evidence on amino acid substitutions indicates that the ongoing evolutionary events occurring among wild ZIKAV lineages in nature may contribute to emerging potential and clinical dynamics.

On the other hand, among non-structural proteins, nucleotide substitutions have been detected (E842D, K859R, A984V, and V1026I) in the NS1 protein of Asian lineage epidemic strains but not in African pre-epidemic strains. Among the NS4B proteins, two amino acid substitutions, V2491 and L2451S, have been detected in the epidemic strains of ZIKAV. NS5 is the largest protein among the virus-encoded proteins, and eight amino acid substitutions, namely T2630V, A2783V, M1970L, K3046R, N2892S, P3158S, D3383N, and S3219D, have been detected in the epidemic Asian lineages but not in the epidemic African lineages.

An analysis of the genome sequences of the Brazilian and African lineages of ZIKAV revealed possible selection pressure events that are exerted at several amino acid positions in the Brazilian epidemic lineages compared to the African lineages of viruses, particularly in the non-structural protein NS4B. This phenomenon might interfere with the phosphorylation of Akt and mTOR, affecting the Akt-mTOR signaling pathway and causing developmental neuropathies in newborns [35].

**Table 1:** Details of mutations in various genes of the ZIKAV genome and their potential effect.

Reference Cited	Location	Substitution	Phenotypic Change
Shan et al. (2020) [33]	Envelop	EV-473M	-Increases neurovirulence -Undergoes maternal-to-fetal transmission -Causes viremia to increase urban transmission
Fontes-Garfias, Camila R 2017 et al. [36]	Envelop	Asn 154	- Mosquito-cell infectivity -Virus assembly
Liu et al. (2021) [37]	Capsid	C-T106A	-Virus fitness advantage -Accelerates the spread in both mosquitoes and rodents -Enhances transmissibility between vectors and hosts
Phumee et al. (2023) [38]	Pre-Membrane	prM-V1A	-Linked with high mortality rate
Yuan et al. (2017) [39]	Pre-Membrane	prM-S17N	-Increased microcephaly in fetus?
Liu et al. (2021) [37]	Pre-Membrane	prM-V123A	-Virus fitness advantage
Yuan et al. (2017) [39]	Pre-Membrane	PrM-S139N	-Accelerates virus infectivity for mouse and human neural progenitor cells -Enhances apoptosis
Xia et al. (2018) [40] Liu et al. (2017) [41]	NS1	NS1-A188V	-Enhances virus infectivity in *Aedes aegypti* -Suppresses Type-T interferon
Liu et al. (2021) [37]	NS1	NS1-A982V	-Virus fitness advantage
Zhang et al. (2023) [42]	NS2A	NS2A-A1204T	-Associated with neurovirulence
Regla Nava et al. (2022) [43]	NS2B	NS2B-139V	-Enhances virus virulence -Escapes from pre-immune dengue antibody
	NS5	NS5-M872	-To be determined
Peng et al. (2022) [44]	NS5	NS5-M114V	-No role in virus replication and transmission potential.
Liu et al. (2021) [37]	NS5	NS5-M3392V	-Virus fitness advantage

## 5. Indian Scenario

In India, evidence of ZIKAV circulation was recorded in the 1950s through a serological survey in which anti-ZIKAV neutralizing antibodies were detected in [12]. However, their public health importance in causing human infections in India is not known or documented.

After the explosive outbreaks of ZIKAV in Brazil in 2013, an eruption of the ZIKAV outbreak occurred in Rajasthan, India, in 2018, affecting 159 individuals, including 63 pregnant women. Between the demonstration of its first activity during the 1950s and its subsequent detection during the 2018 episode in Rajasthan, no virus activity was documented in this country. This could be due to the absence of the virus in circulation or the absence of cases along with the presence of arboviruses in the country, particularly dengue/JE/CHIKV, etc., which have common clinical manifestations that overlap with ZIKAV. Following the episode in Rajasthan, the first laboratory evidence of ZIKAV infection was detected in a pregnant woman from Ahmadabad, Gujarat, India, in 2017 [45]. Its genomic characterization showed the close relationship of the Gujarat isolate with the Malaysian ZIKAV isolate (MYS/P6-740/1966 [KX694533]) recovered in 1966, which indicated the dispersal and silent establishment of the Asian isolate on the Indian subcontinent. Further studies are needed to determine the extent of its distribution and its virological dynamics in vectors and hosts in India. In 2017, a few ZIKAF cases were recorded in a local population, and laboratory evidence was generated from a single case from Krishnagiri district, Tamil Nadu, India. In 2018, outbreaks were subsequently reported in Jaipur, Rajasthan (158 cases), and Madhya Pradesh (127 cases), India. Interestingly, the ZIKAV isolate recovered in Jaipur was found to cluster with the Asian lineage viruses.

After the Rajasthan episode, where three isolates of ZIKAV belonging to the Asian lineage were detected in human cases, about 130 human cases were reported in Madhya Pradesh, India, and a single case was reported in Gujarat (WHO, 2019), which indicated that ZIKAV could spread to other Indian states. In response, countrywide surveillance was conducted against ZIKAV among *Aedes* vectors (79,492 samples (6492 pools) covering 49 districts in high-risk zones in 14 states from 2016 to 2019 [46]). The RT-PCR results revealed the activity of ZIKAV in three pools collected from Jaipur. The detected strains were also clustered among the Asian lineage of ZIKAV, which confirmed the wide circulation of the virus in the country and its ability to cause outbreaks.

In 2021, more than 100 human cases were reported and confirmed to be ZIKAV infections in Uttar Pradesh, India. These cases were confirmed with RT-PCR by detecting the virus-specific genome during the outbreak (https://scroll.in/article/1010555/inside-uttar-pradeshs-zika-outbreak-can-indias-most-populous-state-contain-the-virus-spread accessed on 5 December 2024) [47]. Suspected cases of ZIKAV infection were reported among OPD patients and healthcare workers at private clinics in the Trivandrum area of Kerala, India, in 2021. The complete genome sequence data of two strains (MCL-21-H-8900 and MCL-21-H-8901) recovered from the outbreak were found to be closely related to those of Rajasthan (accession number: MK238037.1), with nucleotide similarities of 99.33% and 99.4%, respectively [48]. However, none of the affected patients from the Trivandrum episode had a history of travel or family members with a history of ZIKAV exposure in a ZIKAV-affected area. Although the source of ZIKAV that infected these patients is not known, the findings strongly suggest the ongoing circulation and silent establishment of ZIKAV in these localities. During the outbreak of ZIKAV in Kerala, Uttar Pradesh, and Maharashtra in 2021, the silent circulation of ZIKAV activity was demonstrated in other states, such as Amritsar, Punjab; New Delhi; Aligarh, Uttar Pradesh; Jodhpur, Rajasthan; Ranji, Jharkhand; Hyderabad, Telangana; and Trivandrum, Kerala.

A recent report on the emergence of a new genotype, i.e., the African genotype of ZIKAV that replaced the circulating genotype in Brazil, alerted public health authorities about possible re-emergence in the country. This finding indicates that the existing genotype of ZIKAV could also be replaced by an emerging new genotype and could lead to outbreaks. The phenomenon of shifting or replacing arboviral strains from one genotype to another has been documented in the cases of dengue, JE, etc. Long-term studies are needed to confirm such events in the case of ZIKAV in India. Countries such as India need proactive efforts in hotspots to track the movement of ZIKAV via improved surveillance tools for timely control.

The available data indicate that ZIKAV is in circulation and is expected to incur new loci, undergo evolution, establish hotspots, and emerge with outbreak potential in this country. Adequate preparedness and strengthening of the surveillance system are essential for mitigating future outbreaks in India.

## 6. Origin and Global Dispersal of ZIKAV

**Figure 4:**
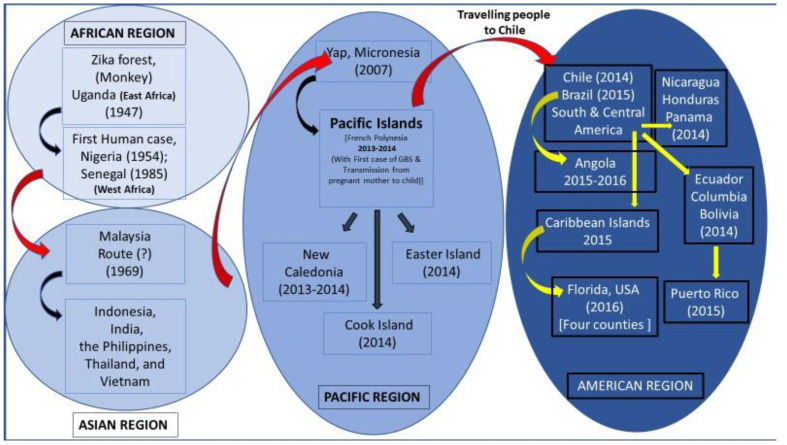
Proposed routes of region-wise global movement of ZIKAV in the African, Asian Pacific, and American regions.

ZIKA virus (ZIKAV) originated in East Africa, and its activity was first detected accidentally in monkeys in Uganda in 1947 [1] during yellow fever (YF) surveillance. It later dispersed to West Africa, as determined by its detection in a human in Nigeria. Simultaneously, it dispersed to Cote d’Ivoire in the 1940s and to Senegal (1985) in the West African region. From Africa, it was introduced and established in Southeast Asia and was in circulation for about 50 years. In Southeast Asia, the virus underwent several evolutionary events for about 50 years and adapted to spread to other parts of the globe. From Southeast Asia, particularly from Cambodia, it spread to French Polynesia, where it caused massive outbreaks from 2013 to 2014. This was confirmed by the close relationship between the Cambodia ZIKAV-2010 strain and the strain that caused the outbreak in French Polynesia (Figure 4).

**Figure 5:**
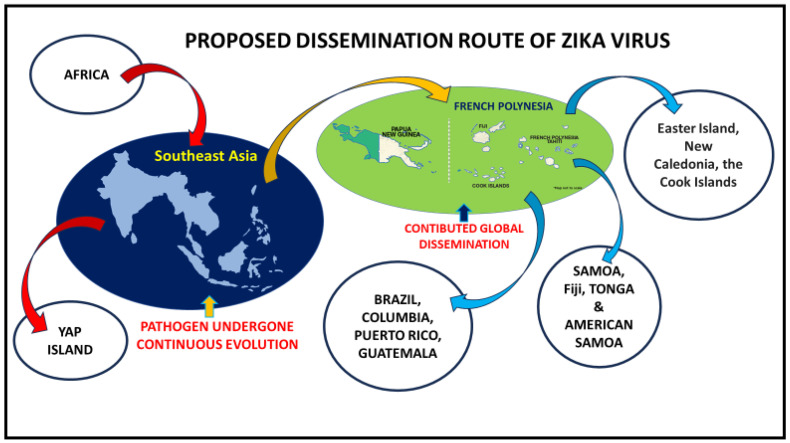
Illustration showing the global dissemination of ZIKAV after undergoing major evolutionary events in Asia and rapid global spread from French Polynesia.

In Southeast Asia, ZIKAV was first detected in Malaysia from field-caught mosquitoes in 1969. The results of a phylogenetic analysis of African and Southeast Asian ZIKAV isolates showed that a geographically distinct lineage of ZIKAV was introduced into SEA regions rather than the African genotype [28]. In 2007, a massive outbreak of ZIKAV was reported from Yap Island, Micronesia; this outbreak affected many people [49]. The virus that caused the outbreak on Yap Island was closely related to the Asian lineage which had been circulating in SEA. This finding highlights the evolutionary, emerging, and dispersal potential of ZIKAV (Figure 5).

The virus probably dispersed to the Pacific Islands, where it caused outbreaks, including those in French Polynesia [50], New Caledonia (2013–2014), the Cook Islands (2014), and the Easter Islands (2014). The unusual phenomenon of GBS and the mother-to-fetal transmission of ZIKAV have been detected in French Polynesia. From the Pacific Islands, particularly from French Polynesia, the virus further spread to the Americas through a single introduction event via the Easter Islands [51], as determined by assessing cases in Brazil in 2015 [4]. The virus subsequently caused outbreaks here in December 2014; in South Ecuador, Colombia, Bolivia, Venezuela, etc., in September 2014; and in Central American countries such as Nicaragua and Honduras, including most of the Caribbean Islands, in April 2015. An outbreak of ZIKAV was reported in Angola in 2015–2016, probably due to the introduction of an Asian lineage of ZIKAV closely related to the one circulating in Brazil in 2015 [52].

During the entire pandemic in the Americas, around 100 million people were infected by the ZIKAV [53], and a strong correlation between ZIKAV infection during pregnancy and fetal congenital malformations was detected [54]. Additionally, following viral infection, severe neurological (encephalitis) and autoimmune (Guillain–Barré syndrome) complications were also recorded [55].

After being introduced to the Americas, ZIKAV cases were reported in Florida in 2016. The virus later dispersed to the four counties of Florida, with most cases observed in Miami-Dade County. The main source of ZIKAV introduction to Florida was predicted to be the Caribbean Islands.

Multiple virus introduction events resulting from the ZIKAV outbreak were also noted in Puerto Rico in 2015–2016. The results of a Bayesian reconstruction analysis of Puerto Rico strains revealed that the outbreak isolates clustered into two clades: Puerto Rico Clade 1 (PRC1) and Puerto Rico Clade 2 (PRC2). PRC1 included viruses originating from South America and the Caribbean Islands, including French Guyana, Brazil, Suriname, the US Virgin Islands, and the Dominican Republic. In contrast, PRC2 included viruses originating from Central America, including Honduras and Nicaragua [56].

ZIKAV (NIV1720741), which was recovered from a patient in Gujarat, India, was speculated to have evolved along with the cluster of strains isolated in Central and South America between 2015 and 2016 [45]. However, further investigation on the full-length sequences of the virus strains is needed for confirmation. Recently, based on NS5 and E gene analysis, ref. [46] proposed a probable separate Indian lineage of ZIKAV that was recovered from Thiruvananthapuram, Kerala, India, which caused an outbreak. However, a complete genome-based analysis is needed to support this claim.

During a recent episode in Pune, Maharashtra, out of 66 confirmed cases of ZIKAF, no severe clinical manifestations of microcephaly were detected among the 26 infected pregnant women (https://www.ndtv.com/india-news/zika-virus-66-cases-reported-in-pune-since-june-including-26-pregnant-women-6279568 accessed on 7 December 2024).

The complete genome sequences of the 197 Asian and African lineages of ZIKAV are available in the global database. However, the limited number of partial genomic sequences of ZIKAV of Indian origin available in the database (Table 2) indicates the need for future attempts to map circulating strains in India.

The above evidence demonstrates the dissemination potential of ZIKAV on the Indian subcontinent and its spread to newer areas. Strengthening virological surveillance is essential to track its movements and clinical outcomes, particularly among pregnant women, for possible severe manifestations.

## 7. Conclusions

ZIKAV is an emerging mosquito-borne virus infection in India. The global isolates of ZIKAV are broadly classified into African and Asian lineages. A detailed analysis of the genomes of virus isolates recovered from different geographical regions showed the continuous process of mutation of structural and non-structural genes in the field isolates of ZIKAV. The detection of ZIKAV in Uttar Pradesh (2019), Gujarat (2019), Tamil Nadu, Kerala (2022), and Karnataka (2023) in India indicates its silent geographical expansion and its emerging potential to spread in the country. The strengthening of surveillance systems should be prioritized to identify hotspots for implementing proactive control strategies.

Global experiences have shown that ZIKAV outbreaks can occur after the virus acquires appropriate mutational changes, which contribute to its virulence, manifestation, and transmissibility characteristics in India. Efforts should also be focused on developing field-adapted quick ZIKAV detection assays for the timely detection of viral antigens in wild vectors to implement vector control efforts. The application of promising unbiased NGS-based methodologies for the rapid detection of ZIKAV should be investigated extensively from an Indian perspective. ZIKAV surveillance needs to be strengthened using advanced tools to determine the magnitude of the problem in the country for adequate public health preparedness.

## Figures and Tables

**Table 2:** Details of the Indian isolates of ZIKAV available in public databases.

Accession no:	Place	Source	Year	Reference
MCL-21-H-8900 and MCL-21-H-8901	Thiruvananthapuram, Kerala, India	Human	2021	Yadav et al. (2022) [48]
MK238037.1	Rajasthan, India	Human	2018	Yadav et al. (2019) [57]
OP678998	Thiruvananthapuram, Kerala, India	Human	2021	Pradeep Kumar et al. (2023) [46]
OP678999	Thiruvananthapuram, Kerala	*Aedes albopictus*	2021	Pradeep Kumar et al. (2023) [46]
OM666892.1	Maharashtra		2021	
NIV1720741/1845ZKV	Gujarat	Human	2016	Sapkal et al. (2017) [45]

## Data Availability

All the data used in the study were obtained from thorough literature surveys and from the ICMR-VCRC Filed Unit Madurai.
